# The Discussion of Ethics

**Published:** 1883-04

**Authors:** 


### The Discussion of Ethics.

The ball has been fully set in motion in the discussion which has been opened in relation to the new code of ethics, adopted by the State Medical Society in 1882, and re-affirmed at its last meeting. The medical press of the State have, until the present time, intentionally ignored the subject from motives of respect, not only to the profession at large, but to the State Society and the American Medical Association, which have been placed in antagonism thereby.

In a late number of the *New York Medical Journal*, an introductory article on " Medical Ethics and Etiquette," from the pen of Dr. Flint Senior, is issued, in which the distinguished writer, in that and successive numbers of that journal, proposes to discuss the code of ethics adopted by the American Medical Association, with commentaries. Following is published the paper on medical ethics, presented to the State Society at its last meeting by Dr. H. R. Hopkins, of this city. The widely distant standpoints from which ethical questions are discussed by these two writers are fair exponents of the diversified opinions held by members of the profession in our own State, and we presume the same diversity of sentiment prevails elsewhere.

Among the objects of the code of ethics is the regulation of the duties of physicians to each other and to the profession at large. Have we not, however, in our zeal to guard the sacred rights of this monopoly in medicine, which we style the regular profession, rather lost sight of the great interests and rights of the public ? We recognize that there may be need of some rules or regulations or laws in relation to the medical profession, and we think that Dr. Worthington Hooker, chairman of the committee on medical education, in his report to the American Medical Association, freely quoted in Dr. Hopkins' paper, hits the nail upon the head when he says that the object of these laws is "to give protection to those *measures* which are calculated to secure to the *community* a well-educated body of physicians." Not much stress is laid here upon the duties of physicians to each other and to the profession at large. The greater duty is to the public. This is a broad position, worthy of the profession and of the representative body before which it was presented, and from which it received an unanimous endorsement.

A code of ethics should be framed upon the principle that the public have rights which the profession are bound to respect; that it is our first duty to present a well-educated and thoroughly trained body of medical men with the greatest latitude in reference to opinions. We must also recognize that until the ranks of the profession are purged of much of the dross which now drags it down to the level of a trade, we cannot afford to criticise our neighbors whom we style irregulars for the lamentable spectacle they present of ignorance, quackery and charlatanry, from the charge of which we ourselves are not altogether innocent.

The logic of events has convinced the younger members of the profession that it is better to have no code than one based upon a foundation which time has undermined, because it failed to fully estimate other rights and prerogatives than those peculiar to medicine alone; and we are inclined to the opinion that our young medical men have caught the spirit and genius of our institutions and have tried to conform therewith a code of ethics which will incorporate, primarily, our duty to the public, and secondarily, our duty to each other. The picture is thus reversed, and we think improved, because it presents a broader outline, on which the responsible duties of the profession can be more clearly portrayed.

The abrogation of the old code has not disturbed our equanimity in the least, for the simple reason that we acknowledge the right of a gentleman, either in or out of the profession, to associate with whom he pleases, and we have not coveted the privilege to trespass beyond the border line of the medical creed in which we were indoctrinated in our pupilage. But we have long since felt that the old code belonged to the past, and we believe that the medical profession should consist of a body of educated and cultivated gentlemen, who would be governed on all ethical questions by those principles of genuine politeness and courtesy observed in refined and cultivated social circles. There is, therefore, an appropriateness to the title, " The Gentleman's Code," given to this late movement in our State Society; and we estimate the medical profession of the Empire State so highly that we think such a code will accord with their instincts and their education.

